# Verification and Comparison of Nine Exhaust Gas Recirculation Mass Flow Rate Estimation Methods

**DOI:** 10.3390/s20247291

**Published:** 2020-12-18

**Authors:** Ádám Nyerges, Máté Zöldy

**Affiliations:** Department of Automotive Technologies, Faculty of Transportation Engineering and Vehicle Engineering, Budapest University of Technology and Economics, H-1521 Budapest, P.O.B. 91, Hungary; mate.zoldy@gjt.bme.hu

**Keywords:** diesel engines, dual loop exhaust gas recirculation, mass flow rate estimation, air path system sensors

## Abstract

Modern Diesel engines have complex exhaust gas recirculation (EGR) systems. Due to the high temperatures, it is a typical issue to measure EGR mass flow rates in these complex control systems. Therefore, it is expedient to estimate it. Several sensed values can help the estimation: the fresh air mass flow rate, the fuel consumption, pressures, temperatures and mass fractions in the air path system. In most of the articles, the EGR mass flow rate estimation is done by the pressures. However, gas composition based models usually would be better for control aims. In this paper, nine EGR estimation methods will be presented: an important outcome is to present the required sensor architectures and estimation challenges. The comparison will be made by measurement results both in stationary operation points and transient cycles. The estimated EGR mass flow rates will be evaluated by verification conditions. The results will prove that the intake and exhaust side oxygen sensors can give verifiable signals for EGR mass flow rate estimation. In contrast, the applied fresh air mass flow rate and the nitrogen-oxide signals are not accurate enough to provide verifiable EGR mass flow rates in every operating condition. The effects of sensor inaccuracies will also be considered.

## 1. Introduction

Due to the more and more strict emission regulations [1], internal combustion engines need more complex systems. In the emission reduction systems, exhaust gas recirculation (EGR) systems have an important rule, typically in Diesel engines.

Although the conventional powertrain systems nowadays seem to be ended, Diesel engines will probably be used in commercial vehicles for a long time. The engines are different from the passenger cars’ engines. They have a narrower speed range and a large torque range. From the modeling aspect, the smaller speed range is an advantage. Due to the periodic operation, several parameters change effectively in the engine speed function—for example, the volumetric efficiency can have a significant change. A smaller speed range achieves that several parameters can be simplified to constants [2,3]. Thus, commercial vehicles’ engines’ modeling needs a different approach and has new research opportunities in model simplification [4].

In our university research, we develop engine models [5] and develop control strategies [6]. One of our future aims is to create a new experimental engine model for the dual loop EGR system with three model states. For this aim, it is necessary to estimate the EGR mass flow rates for the engine’s control inputs. For the validation, measurements are needed – and the main issue of this paper appeared at this phase.

Due to accuracy and validation problems, finally, nine EGR estimation methods were made. The estimation methods will be classified by their theoretical basics. As subtopics, the engine mass flow rate estimation and gas composition mixing estimation opportunities also will be considered. The engine mass flow rate can be estimated by its fuel and oxygen consumption or by its boost pressure, speed and volumetric efficiency. Gas composition mixing equations can be based on oxygen and nitrogen oxide mass fractions. Besides, the fresh air mass flow meter’s signal can be used to simplify the estimation methods. Finally, the intake-exhaust pressure difference based methods are much different and simpler from the others.

As will be presented, there are significant differences between the estimated mass flow rates. Each method can probably be tuned for better accuracy with high work effort but now, a fast solution is necessary for further work for our research. These tuning opportunities will also be presented. The main challenge of the evaluation is the unknown exact EGR mass flow rate. The estimation results only can be evaluated by their logical tendencies and they only can be compared to each other. Thus, the comparison will use verification conditions and the nine methods will be evaluated by the analysis of the estimated results’ tendencies.

For our further research, the detailed analysis of the sensors’ inaccuracies is not important. Therefore this paper will not consider the propagation of uncertainty.

The final result will probably be useful for other researchers on the same topic to find a better way for EGR mass flow rate estimation. Moreover, perhaps, the collection of the different methods also can give new ideas for the readers.

## 2. Exhaust Gas Recirculation

Diesel engines typically have turbochargers. An up-to-date engine is equipped with a dual loop EGR system, for example, they have a high pressure (HP) EGR on the high pressure side of the turbocharger (short loop) and they have a low pressure (LP) EGR on the low pressure side of the turbocharger (long loop). Both of these systems can be supported with exhaust brakes to reach very high EGR rates [7]. In a modern engine these flaps are controlled electronically. The EGR systems have several advantages but they can also change the engine’s performance in the wrong direction. For example, with EGR the nitrogen-oxide emission can be extremely reduced [8,9]. At the same time, the soot emission can increase as well [10,11]. Besides, both EGR systems can decrease fuel consumption but higher EGR amounts always increase it—several optimal operation points could be found by an appropriate control system [12].

The control synthesis for dual loop EGR systems is an up-to-date research area in the last decade [2,4,5,13,14,15,16,17,18]. The first step in controlling is usually the modeling of the system. Engine models can be based on several physical properties in the air path system. One typical modeling type is based on the pressures and mass flow rates [2,3,15]. In these models, the EGR mass flow rate can be estimated by the orifice equation between the exhaust and the intake side [2]. Other engine models are based on the composition changes in the air path system [13,16]: air fraction, burned gas fraction, oxygen fraction and so forth. Either type is utilized, usually the EGR mass flow rate should be estimated somehow.

In most articles, the EGR mass flow rate is estimated by the pressure difference between the exhaust and intake sides. There are several articles where the orifice equation is validated and tuned for this aim [19,20,21]. The composition change estimation is much rare [22,23,24].

The EGR mass flow rate measurement and estimation issue always go together with the same mass flow rate issue through the cylinders. From another aspect, there are two effects in the air path system that determine the realized EGR parameters. The mass flow rate and the composition change through the engine generate the pressure, temperature and composition differences between the exhaust and the intake side. The other effect that changes these parameters is mixing the fresh air and the recirculated exhaust gas in the EGR mixer. By the equations of these processes, the EGR mass flow rate can be determined.

Due to the relatively high temperatures, the engine’s mass flow rate is also difficult to measure. In most of the articles, there are two types of engine mass flow rate estimations. The first one considers the cylinders’ air consumption with the speed, the displacement, the intake air density and the volumetric efficiency [2,3]. The other one determines the engine mass flow rate by the oxygen fraction change between the intake and the exhaust side [23,25].

The fresh air and the exhaust gas mixing on the intake side also can be modeled in several ways. In this process, there are similar changes: pressure, temperature and composition. By the pressures, the processes can be estimated with the orifice equations [19]. For composition change equations, the air fraction or the burnt gas fraction are typically used [22]. Besides, the oxygen and NO_x_ fractions also can be measured for validation [26].

## 3. Measurement System

For the measurements, an engine dyno was used, equipped with a medium duty Diesel engine. This engine was included in several articles [2,3,5,6,23,26,27]. The operation of the engine was modelled in several ways, for example in Reference [26] a detailed engine model was published, where the burn rates, the friction loads and also the pressures and the temperatures in the air path system were validated. In Reference [2] and in Reference [5] control algorithms were developed for the HP EGR system. In Reference [27] a detailed injector model was presented. Nowadays, the engine is also tested with biofuels [28].

The main parameters of the engine can be seen in Table 1.

The engine is equipped with a dual loop EGR system. Both of the EGR systems are supported by exhaust brakes, for example, the EGR systems can be supported separately. The HP EGR systems supporter exhaust brake is mounted downstream the turbine, the LP EGR system’s one is mounted downstream the LP EGR branching.

The pressures and the temperatures can be measured upstream of the compressor and downstream the intercooler on the intake side. They can also be measured on the exhaust manifolds exhaust side and downstream the first exhaust brake. Here the DPF (Diesel particulate filter) can generate a pressure drop. To avoid validation problems, we also did measurements without DPF. The validation of these pressure and temperature sensors was presented in Reference [26].

The oxygen and nitrogen-oxide measurement can be done in the intake manifold and downstream the first exhaust brake. On the intake side there are two sensors: a Lambda sensor and a nitric oxide sensor. On the exhaust side only a nitric oxide sensor was used (our nitric oxide sensor can measure both the oxygen and the NO*_x_* fractions). The validation of the nitric oxide and oxygen sensors was presented in Reference [26].

An air mass flow rate sensor is also applied upstream of the LP EGR outlet. The validation of the air mass flow rate sensor was also presented in Reference [26]. However, there were some modification in the intake side since then, therefore a signal check will also be done in this paper.

The fuel consumption of the engine can be measured in two ways. Once the engine dyno has a gravimetric fuel measurement system. The signal of this system sometimes difficult to use. For instance, during the refilling, there is not any information from it. However, from the ECU of the engine the target injected fuel mass also can be reached. Finally, with the more accurate gravimetric fuel measurement system, the ECU’s fuel dose command was validated and used for the research by Reference [26]. The ECU based fuel consumption measurement has some fluctuation caused by the engine dyno’s control system—we could not eliminate this property.

The details of the applied sensors can be seen in Appendix C.

**Figure 1:**
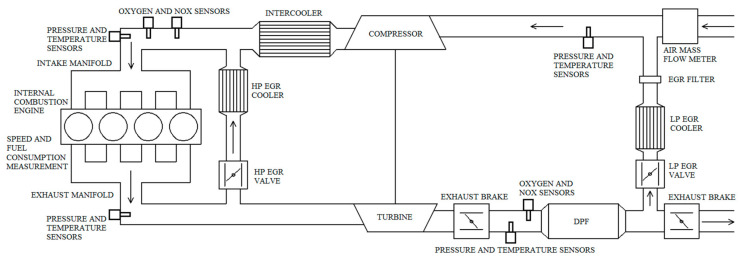
Layout of the air path system [2].

The layout of the air path system can be seen in Figure 1.

The list of the sensors and their types can be seen in Table 2.

## 4. Estimation Aspects

In this chapter, the theoretical basics will be presented for the nine methods. From these basics, the equations for the EGR mass flow rate estimation can be created.

### 4.1. Pressures in the EGR Systems

The EGR systems can be modeled as orifices. The EGR flap valve is the controllable area of the EGR pipe. By opening the EGR flap valve, its discharge coefficient also changes. The real area and the discharge coefficient determines the effective area of the orifice. By these, the mass flow rate can be estimated by the well-known orifice equation [4]. Between two chambers with different pressures, the orifice equation can be written as follows:

(1) $$\pi \leq \pi_{crit}\Rightarrow \sigma_{orifice}=A_{eff}\frac{p_{high}}{\sqrt{RT_{high}}}\sqrt{\frac{2p_{low}}{p_{high}}\left(1-\frac{p_{low}}{p_{high}}\right)}$$

(2) $$\pi ≻\pi_{crit}\Rightarrow \sigma_{orifice}=A_{eff}\frac{p_{high}}{\sqrt{RT_{high}}}\frac{1}{\sqrt{2}}.$$

The advantage of the orifice equation is the relatively easy validation. It is only necessary to measure the pressures and the intake temperature to estimate the mass flow rate. Pressure and temperature sensors can be appropriate accurate in their typical value intervals in engine air path systems. However, determining the effective area can be problematic because, due to the flow area geometry and the turbulences, it is not the same as the measurable geometric area. The discharge coefficients were validated in Reference [26] by the pressure sensors. In this paper, it will be better to handle the discharge coefficients as tunable parameters in the orifice equation. The tuning will be mentioned in the measurement evaluation.

The momentary effective area is calculated by the EGR valve actuator’s command opening percentage.

The orifice equation may give inaccurate results if the pressure difference is small between the two volumes because the difference between the measured pressures can be smaller than the absolute tolerance of the sensors. In this case, the relative inaccuracy of the pressure sensors causes inaccurate results.

### 4.2. Mass Flow Rate through the Cylinders

The mass flow rate through the cylinders can be estimated by the air consumption of the cylinders [2]. The engine speed and the engine’s displacement determine a theoretical mass flow rate. The boost pressure can increase it by the increased intake air density. The volumetric efficiency usually is a function of engine speed. In commercial vehicles, the engine speed range is smaller, for example, the volumetric efficiency can be modeled as a constant [3].

(3) $$\sigma_{eng}=\eta_{vol}\frac{p_{2}}{RT_{2}}\frac{V_{d}n_{eng}}{2}.$$

Equation (3) is tunable by the volumetric efficiency.

### 4.3. Component Fraction Changes on the Intake Side Due to EGR

Equation (4) presents the mixing of the fresh air and the recirculated exhaust gas in the intake side from the composition change aspect [23,29]. For the EGR mass flow rate estimation, it is necessary to know the component mass fraction of the intake fresh air, exhaust gas and mixed gas. It is also required to measure the intake fresh air mass flow rate. As can be seen, Equation (4) is suitable on its own for EGR mass flow rate estimation. It is only necessary to choose the measurable component mass fraction of the gases.

(4) $$\sigma_{EGR}=\frac{x_{0}-x_{2}}{x_{0}-x_{3}}\sigma_{air0}=\frac{x_{0}-x_{2}}{x_{0}-x_{3}}\left(\sigma_{eng}-\sigma_{EGR}\right).$$

If the instantaneous intake fresh air mass flow rate is not reachable, the estimated engine mass flow rate also can be used. If the distance between the mass flow meter and the engine is neglected, the fresh air mass flow rate can be estimated as the difference between the engine mass flow rate and the EGR mass flow rate. This is presented at the end of Equation (4) and in Equation (5), it is rearranged to estimate the EGR mass flow rate in another way.

(5) $$\sigma_{EGR}=\frac{x_{0}-x_{2}}{2x_{0}-x_{3}-x_{2}}\sigma_{eng}.$$

How can be the component mass fraction based estimations inaccurate? Usually, when the changes are too small during the gas mixing. For instance, if a very small amount of EGR is applied, the oxygen fraction change will also be minimal. In this case, the sensors’ inaccuracy may result inaccurate estimation. For example when a small amount of EGR is applied the oxygen mass fraction change between *x*_0_ and *x*_2_ can be as small as the absolute tolerance of the sensors. for example, the nominator of Equation (5) is sensitive with small EGR rates which can occurs inaccuracy.

With nitrogen-oxide measurement, the inaccuracy can appear with high EGR rates. For instance, when a huge amount of EGR is applied, the nitrogen-oxide fraction can be reduced below 20–30 ppm [11]. This quantity can reach the nitric oxide sensors measurement range and it may result in inaccurate estimation.

### 4.4. Component Fraction Changes Due to the In-Cylinder Combustion Processes

If the instantaneous fuel consumption is known, the oxygen consumption of the engine can be estimated (by assuming a perfect combustion process). Thus, the oxygen mass flow rate decreases with the oxygen consumption from the intake to the exhaust side due to the chemical reactions. With EGR the oxygen concentration on the intake side is less than in the fresh air. Considering these, the oxygen mass fraction can be estimated in the exhaust manifold as follows [23]:

(6) $$x_{O_{2}eng}\approx x_{O_{2}3}=x_{O_{2}2}-\frac{x_{O_{2}0}K_{L0}\sigma_{f}}{\sigma_{eng}}.$$

Equation (6) can be rearranged in the following way, where the engine mass flow rate can be measured with a new equation [23]:

(7) $$\sigma_{eng}=\frac{x_{O_{2}0}K_{L0}\sigma_{f}}{x_{O_{2}2}-x_{O_{2}3}}.$$

The accuracy of Equation (7) depends on the oxygen consumption of the engine. With low oxygen consumption, accuracy becomes worse. When there is no fuel consumption, the numerator and the denominator also converge to zero—in these engine operations, the estimation may be inaccurate.

### 4.5. Mass Flow Rates in the EGR Mixer

As it was presented in Section 4.3, a simple equation can be prescribed between the intake fresh air mass flow rate, the engine mass flow rate and the EGR mass flow rate. When exhaust gas is recirculated in the air path system, the intake fresh air mass flow rate will decrease because the change of the engine mass flow rate is usually not so significant (for instance, with HP EGR the boost pressure can change). In this case, Equation (8) expresses the mass conservation law. Due to the long distance between the EGR mixer and the equation of the cylinder’s mass flow rate (8) is only accurate in stationary engine operation. However, usually, this distance can be neglected.

(8) $$\sigma_{EGR}=\sigma_{eng}-\sigma_{air0}.$$

With low EGR rates, the difference between the fresh air mass flow rate and the engine mass flow rate is very low. The engine mass flow rate estimation accuracy and the mass flow meter’s measurement accuracy may cause significant inaccuracy.

The turbulence upstream of the mass flow meter can make it inaccurate. Therefore the measured signal is handled as a tunable parameter. Former experiences will be used for the setting of the signal from Reference [26]. Besides, there will be a simple check for the tuning.

## 5. EGR Mass Flow Rate Estimation Methods

By the basic equations in Section 4, nine estimation methods can be created for the EGR mass flow rate. The classification of these methods can be seen in Table 3 and Appendix A. The serial numbers of the methods will be important in the following chapters because this is the shortest way to refer to them. Section 5 presents the nine estimation methods.

### 5.1. 1st Method: Estimation by the Engine Volumetric Efficiency and by the Oxygen Mass Fraction Changes

Putting the engine mass flow rate from Equation (3) into Equation (5) with oxygen mass fractions results in the following equation:

(9) $$\sigma_{EGR}=\frac{x_{O_{2}0}-x_{O_{2}2}}{2x_{O_{2}0}-x_{O_{2}3}-x_{O_{2}2}}\eta_{vol}\frac{p_{2}}{RT_{2}}\frac{V_{d}n_{eng}}{2}.$$

Required measured signals:

- intake side oxygen mass fraction,
- exhaust side oxygen mass fraction,
- intake manifold pressure,
- intake manifold temperature,
- engine speed.

### 5.2. 2nd Method: Estimation by the Engine Fuel Consumption and by the Oxygen Mass Fraction Changes

Putting the engine mass flow rate from Equation (7) into Equation (5) with oxygen mass fractions results in the following equation:

(10) $$\sigma_{EGR}=\frac{x_{O_{2}0}-x_{O_{2}2}}{2x_{O_{2}0}-x_{O_{2}3}-x_{O_{2}2}}\frac{x_{O_{2}0}K_{L0}\sigma_{f}}{x_{O_{2}2}-x_{O_{2}3}}.$$

Required measured signals:

- intake side oxygen mass fraction,
- exhaust side oxygen mass fraction,
- fuel consumption.

### 5.3. 3rd Method: Estimation by the Intake Fresh Air Mass Flow Rate and by the Oxygen Mass Fraction Changes

Expressing Equation (4) with oxygen mass fractions results in the following equation:

(11) $$\sigma_{EGR}=\frac{x_{O_{2}0}-x_{O_{2}2}}{x_{O_{2}0}-x_{O_{2}3}}\sigma_{air0}.$$

Required measured signals:

- intake side oxygen mass fraction,
- exhaust side oxygen mass fraction,
- intake fresh air mass flow rate.

### 5.4. 4th Method: Estimation by the Engine Volumetric Efficiency and by the NO_x_ Mass Fraction Changes

Putting the engine mass flow rate from Equation (3) into Equation (5) with nitrogen-oxide mass fractions results in the following equation:

(12) $$\sigma_{EGR}=\frac{x_{NO_{x}0}-x_{NO_{x}2}}{2x_{NO_{x}0}-x_{NO_{x}3}-x_{NO_{x}2}}\eta_{vol}\frac{p_{2}}{RT_{2}}\frac{V_{d}n_{eng}}{2}.$$

Required measured signals:

- intake side nitrogen-oxide mass fraction,
- exhaust side nitrogen-oxide mass fraction,
- intake manifold pressure,
- intake manifold temperature,
- engine speed.

### 5.5. 5th Method: Estimation by the Engine Fuel Consumption and by the NO_x_ Mass Fraction Changes

Putting the engine mass flow rate from Equation (7) into Equation (5) with nitrogen-oxide mass fractions results in the following equation:

(13) $$\sigma_{EGR}=\frac{x_{NO_{x}0}-x_{NO_{x}2}}{2x_{NO_{x}0}-x_{NO_{x}3}-x_{NO_{x}2}}\frac{x_{O_{2}0}K_{L0}\sigma_{f}}{x_{O_{2}2}-x_{O_{2}3}}.$$

Required measured signals:

- intake side nitrogen-oxide mass fraction,
- exhaust side nitrogen-oxide mass fraction,
- intake side oxygen mass fraction,
- exhaust side oxygen mass fraction,
- fuel consumption.

### 5.6. 6th Method: Estimation by the Intake Fresh Air Mass Flow Rate and by the NO_x_ Mass Fraction Changes

Expressing Equation (4) with nitrogen-oxide mass fractions results in the following equation:

(14) $$\sigma_{EGR}=\frac{x_{NO_{x}0}-x_{NO_{x}2}}{x_{NO_{x}0}-x_{NO_{x}3}}\sigma_{air0}.$$

Required measured signals:

- intake side nitrogen-oxide mass fraction,
- exhaust side nitrogen-oxide mass fraction,
- intake fresh air mass flow rate.

### 5.7. 7th Method: Estimation by the Engine Volumetric Efficiency and by the Intake Fresh Air Mass Flow Rate

Putting the engine mass flow rate from Equation (3) into Equation (8) with oxygen mass fractions results in the following equation:

(15) $$\sigma_{EGR}=\eta_{vol}\frac{p_{2}}{RT_{2}}\frac{V_{d}n_{eng}}{2}-\sigma_{air0}.$$

Required measured signals:

- intake manifold pressure,
- intake manifold temperature,
- engine speed,
- intake fresh air mass flow rate.

### 5.8. 8th Method: Estimation by the Engine Fuel Consumption and by the Intake Fresh Air Mass Flow Rate

Putting the engine mass flow rate from Equation (3) into Equation (8) with nitrogen-oxide mass fractions results in the following equation:

(16) $$\sigma_{EGR}=\frac{x_{O_{2}0}K_{L0}\sigma_{f}}{x_{O_{2}2}-x_{O_{2}3}}-\sigma_{air0}.$$

Required measured signals:

- intake side oxygen mass fraction,
- exhaust side oxygen mass fraction,
- fuel consumption,
- engine speed,
- intake fresh air mass flow rate.

### 5.9. 9th Method: Estimation by the Pressure Drop on each EGR System

Expressing Equation (1) for each EGR systems results in the following equations:

(17) $$\sigma_{HPL}=A_{HPL}\frac{p_{3}}{\sqrt{RT_{3}}}\sqrt{\frac{2p_{2}}{p_{3}}\left(1-\frac{p_{2}}{p_{3}}\right)}\geq 0$$

(18) $$\sigma_{LPL}=A_{LPL}\frac{p_{5}}{\sqrt{RT_{5}}}\sqrt{\frac{2p_{1}}{p_{5}}\left(1-\frac{p_{1}}{p_{5}}\right)}\geq 0.$$

Required measured signals:

- intake manifold pressure,
- exhaust manifold pressure,
- pressure upstream the compressor,
- pressure downstream the turbine,
- exhaust manifold temperature,
- temperature downstream of the turbine.

## 6. Measurement Results

With ideal, accurate sensors, evaluating the different methods would be easy since the estimation signals would be nearly identical. However, due to the sensors’ inaccuracy and the simplifications of the methods, there will be differences between the results. Thus, the evaluation is a challenge because the exact EGR mass flow rate is unknown: the estimation results only can be compared to each other.

The following verification aspects were considered during the evaluation:

- with closed EGR valves there is not any EGR mass flow rate,
- with open EGR valves and more and more closed supporter exhaust brakes the EGR mass flow rate should increase (as far as the engine mass flow rate does not decrease),
- lower signal fluctuation is better,
- in the transient during between two operation points the new mass flow rate balance emerges in a few seconds (it is audible near the engine).

The handling of the tunable parameters:

- engine volumetric efficiency: the former value was considered from References [2,3]. Its value for the whole speed range is 0.9,
- air mass flow meter value: due to smaller modifications in the intake pipes it had to be re-validated. It was validated by the volumetric efficiency based engine mass flow rate estimation. The result will be able to see in Figure 8,
- effective maximum area of the EGR valve: the parameter is a multiplier in the orifice equation. The result will be present that the maximum effective area of the EGR valves cannot solve the issue of the pressure based EGR mass flow rate estimation.

In the presented operation points and cycles these parameters are not modified.

The comparison’s main goal is the verification of the nine methods. The methods will be classified into the following categories:

- non-verifiable, not recommended methods,
- verifiable but due to sensor inaccuracy not applicable for controlling aims,
- verifiable but recommended for further research to make it more accurate,
- verifiable and recommended for controlling aims.

### 6.1. Experiences in Stationary Operation Points

In previous articles, several parameters in the measurement system were validated [16]. With these experiments in the first phase of the evaluation, the tunable parameters were set again for more accurate results. In the first phase, the measurements are done in stationary operation points with opened EGR valves and gradually closed exhaust brakes. These measurements are done in several engine speeds and torques. The speeds and torques are chosen by the typical ranges of the WHSC (World Harmonized Stationary Cycle) and the WHTC (World Harmonized Transient Cycle) regulations [1]. In this article, two operation points will be presented: 1250 L/min with 50 Nm (lower speed and torque) and 1750 L/min with 200 Nm (higher speed and torque). Both EGR systems were used. Some other results can be seen in Appendix B.

**Figure 2:**
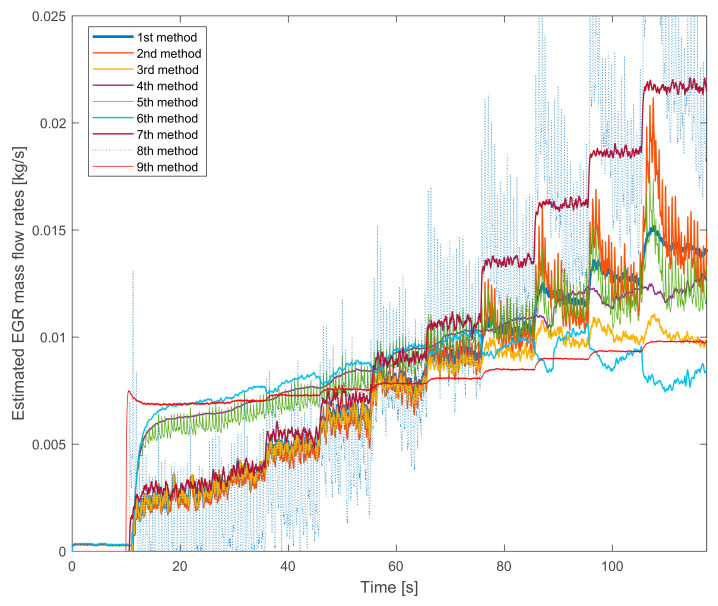
The estimated EGR mass flow rates with gradually closed exhaust brake. The HP EGR valve is opened, the engine runs on 1250 L/min and 50 Nm.

Figure 2 shows the estimated EGR mass flow rate signals with HP EGR on the lower speed and torque. The first consequences can be drawn: there are significant differences between the signals.

The 8th method’s signal is depicted with a pale line because its significant fluctuation would make the figure with a thicker line incomprehensible. Apart from the big fluctuation, its average value also different from the other methods’ values. At a lower EGR rate it underestimates the others. On higher EGR rates it overestimates the other signals. The reason for the high fluctuation will be explained in further chapters.

The 7th method’s signal does not significantly fluctuate but the average value is similar to the 8th method’s signal. The similar theoretical approach causes it: both of them are based on the difference between the engine mass flow rate and the fresh air mass flow rate.

Another interesting tendency can be seen on the signal of the 9th method, which uses the orifice equation. This signal has the lowest fluctuation. As can be seen, on lower EGR rates it overestimates the others. On higher EGR rates it underestimates the other signals. By the tuning of the effective area just an offset can be changed with this curve, for example, it is not possible to change the slope of the curve. Its shape will always different from the other curves.

Besides, the similar theoretical basics also can be seen on the other curves. The tendency of the 1st and the 4th methods, the tendency of the 2nd and the 5th methods and the tendency of the 3rd and the 6th methods are similar (the 7th and the 8th method have already been mentioned).

**Figure 3:**
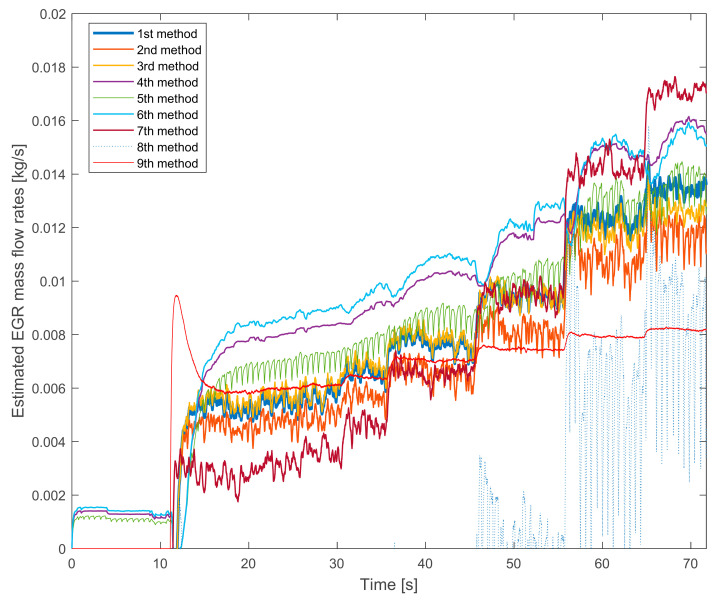
The estimated EGR mass flow rates with gradually closed exhaust brake. The HP EGR valve is opened, the engine runs on 1750 L/min and on 200 Nm.

In Figure 3 the same measurements are shown on the higher speed and torque. The 8th method’s signal is unusable again. On lower EGR rates it gives a negative signal. As the tendency of the 9th method’s signal is also similar.

The differences are more significant than in Figure 2 were. The difference between the lowest and the highest values is more than twofold.

The pairs that were able to see in Figure 2 are changed in Figure 3. Here the tendencies that are similar:

- the 1st and the 3rd methods,
- the 4th and the 6th methods.

The common is that these pairs use the same mass fractions in their estimation.

Partially the 2nd and the 5th methods are also similar. Their fluctuations are higher. The reason is that they are based on the fuel consumption measurement, which also has a significant fluctuation.

The 7th method’s signal is different from the others again.

**Figure 4:**
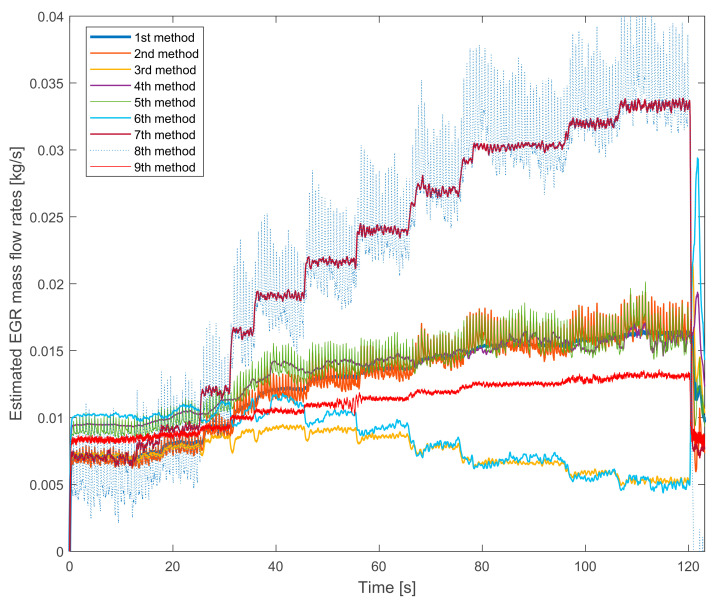
The estimated EGR mass flow rates with gradually closed exhaust brake. The LP EGR valve is opened, the engine runs on 1250 L/min and on 50 Nm.

The measurements with LP EGR system presents new properties of the estimation methods. Figure 4 shows the results on the lower speed and torque.

The signal pairs shown in Figure 2 appear again—probably due to the similar torque, which means small boost pressure. The properties of the 7th, the 8th and the 9th methods are similar as they were earlier.

However, there is a new phenomenon in the behavior of the 3rd and the 6th method’s signals. On higher EGR rates, they start to decrease, which certainly a false tendency—with more and more closed exhaust brake the EGR mass flow rate should not decrease. The increase of the pressure based estimation also proves it.

**Figure 5:**
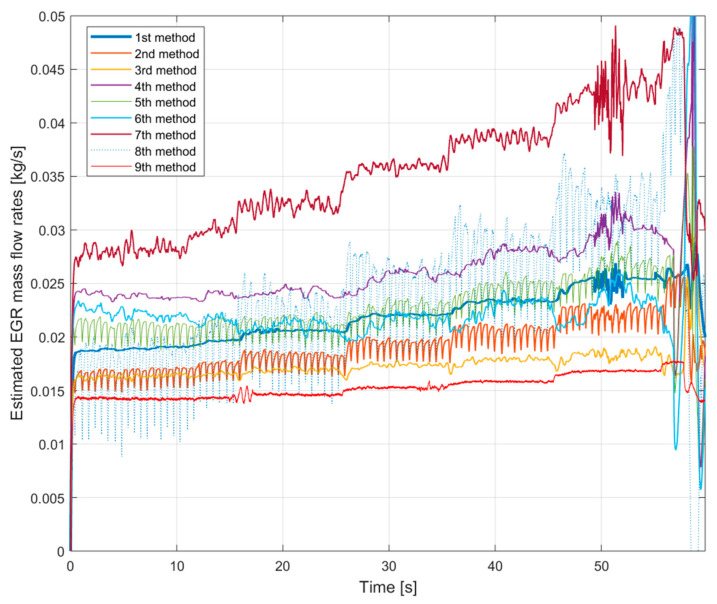
The estimated EGR mass flow rates with gradually closed exhaust brake. The LP EGR valve is opened, the engine runs on 1750 L/min and on 200 Nm.

Finally, in this chapter the LP EGR measurement signals can be seen on the higher speed and torque. The applicable maximum EGR mass flow rate is not so high in this operation point because the engine reaches the stoichiometric air-fuel ratio (e.g., the exhaust gases run out of oxygen). The unstable operation causes the fluctuating shape of the curves at the end of Figure 5 with too high EGR rates, for example, the engine nearly stopped.

The formerly presented pairs also can be seen here. They are recognizable by the values and the shapes of the curves. As in Figure 3. offsets appeared between the pairs.

Summarizing the verification experiences with the stationary operation points:

- the 8th method is not suitable for EGR mass flow rate estimation because it is not verifiable,
- the 7th method’s values are similar to the 8th method’s ones and it is much different from the other signals,
- the pressure drop based 9th method has a lower slope than the others,
- the fuel consumption based 2nd, 3rd and 8th methods have higher fluctuations due to the fluctuation of the fuel consumption measurement,
- on lower EGR rates the NO_x_ mass fraction based 4th, 5th and 6th methods estimate higher values than the oxygen mass fraction based 1st, 2nd and 3rd methods.

### 6.2. Experiences in Transient Cycles

The long term goal of the research is emission control by EGR systems. Regulations prescribe the emission of internal combustion engines. Therefore, the EGR mass flow rate estimation measurements were also evaluated in the EU legally mandated World Harmonized Transient Cycle [1].

**Figure 6:**
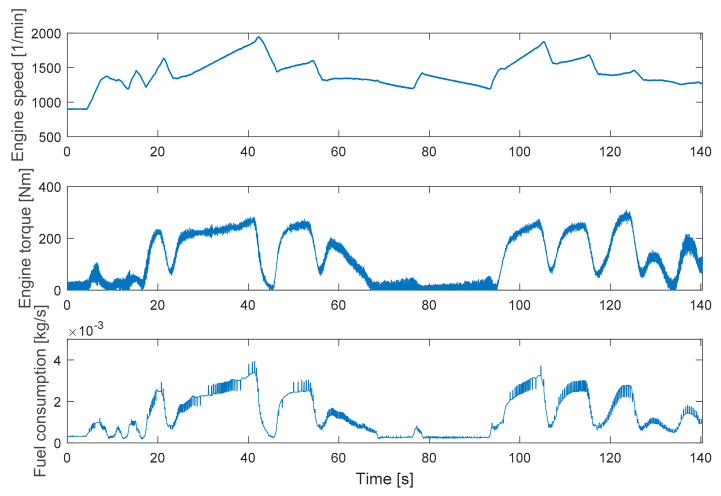
The applied section of the World Harmonized Transient Cycle (WHTC) without EGR: speed, torque and fuel consumption.

For the evaluation in transient cycles, a representative part of WHTC was chosen. From 1190 to 1330s several different operation points can be found: idle, high load on low revs and low loads on higher revs with slow and fast changes. Negative loads were not used because the applied eddy current brake cannot produce negative torques. The selected period is depicted in Figure 6.

Besides, on higher loads with EGR the engine can reach its air-fuel ratio limit. To avoid very low air-fuel ratios, the WHTC torque demand was modified to 50%, compared to the original torque demand.

In Figure 6 the measurement disturbances also can be seen on the fuel consumption signal.

**Figure 7:**
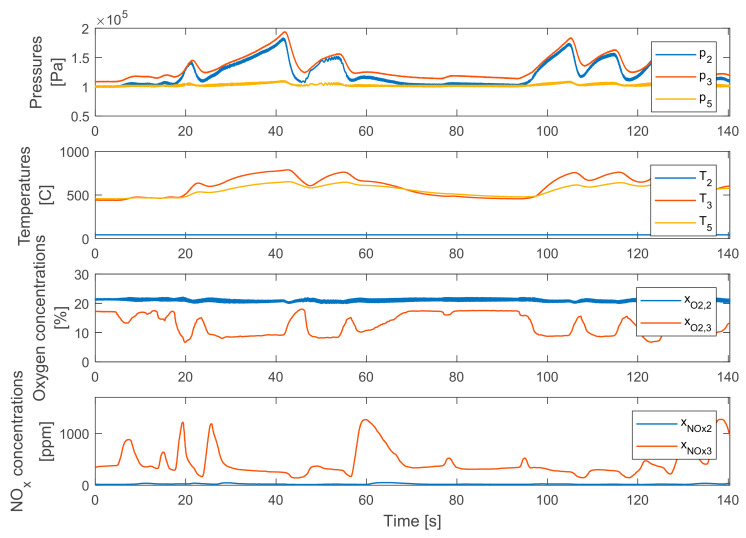
The achieved pressures, temperatures, oxygen and nitrogen-oxide mass fractions during the applied WHTC section.

Figure 7 shows the EGR mass flow estimation methods main input parameters in the same WHTC period. These are the typical values. In this measurement the EGR valves were closed.

**Figure 8:**
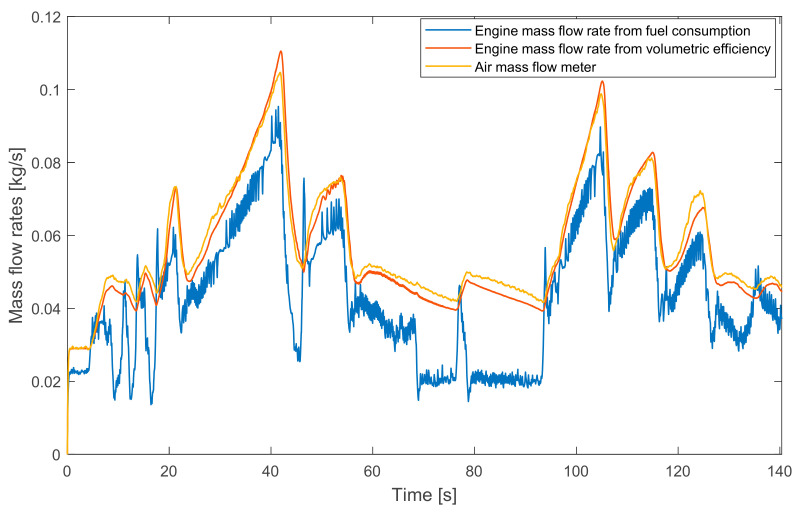
Comparison between the measured intake fresh air mass flow rate and the estimated engine mass flow rates without EGR.

In Section 4 two equations were presented for the estimation of the cylinders’ mass flow rate. These were Equation (3) and Equation (7). If the EGR valves are closed, the value of the fresh air mass flow rate is the same but it overtakes the estimations due to its place in the air path system. In this case, the three values can be compared to each other. This can be seen in Figure 8. 

Figure 8 shows that the signal of the intake fresh air mass flow rate and the estimated engine mass flow rate run close to each other. The reason for this similarity is the appropriate setting of the tunable parameters: setting the engine volumetric efficiency and tuning the measured fresh air mass flow rate signal [2]. As explained previously, the oxygen mass fraction based engine mass flow rate estimation can be inaccurate on low loads. In Figure 8, this property appears at several intervals. Therefore, the EGR mass flow rate estimation on low loads can be inaccurate.

**Figure 9:**
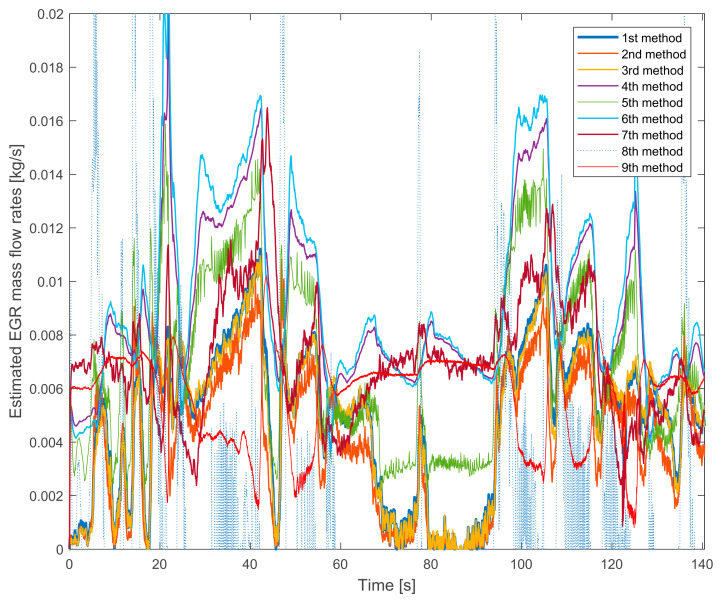
The estimated EGR mass flow rates during the applied WHTC cycle with opened HP EGR valve.

In engine control, the estimation should give usable signals in transient cycles. In Figure 9, the HP EGR mass flow rate estimations can be seen in the presented WHTC interval. During the measurement, only the HP EGR valve was open. The supporter exhaust brakes do not affect here. The differences between the estimation are significant both in low and high loads.

Even so, the previously observed properties also can be seen here: the oxygen mass fraction based methods gives similar results such as the nitrogen-oxide based methods. The last three methods are much different again.

**Figure 10:**
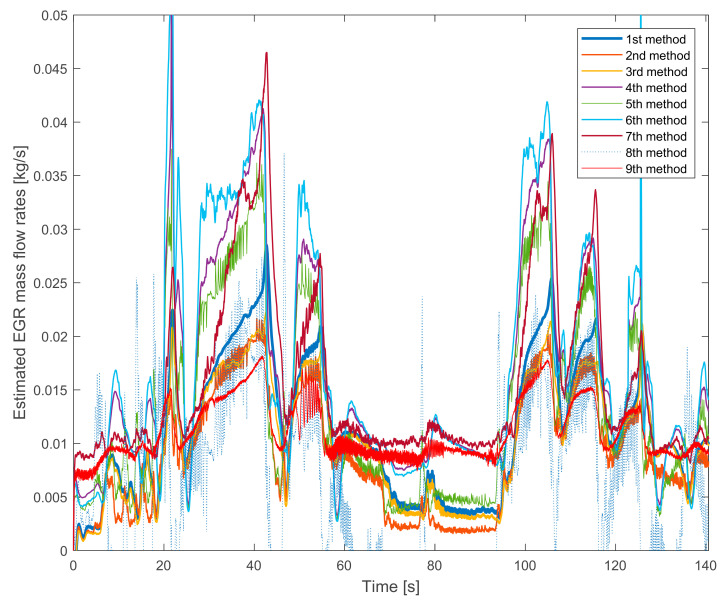
The estimated EGR mass flow rates during the applied WHTC cycle with opened LP EGR valve.

Compared to the HP EGR measurements, the LP EGR results are more unified. There are smaller differences between the tendencies. These are shown in Figure 10. Even the 9th method’s signal follows better the other signals too.

The nitrogen-oxide mass fraction based signals consequently have two times bigger values than that the oxygen mass fraction based signals have.

**Figure 11:**
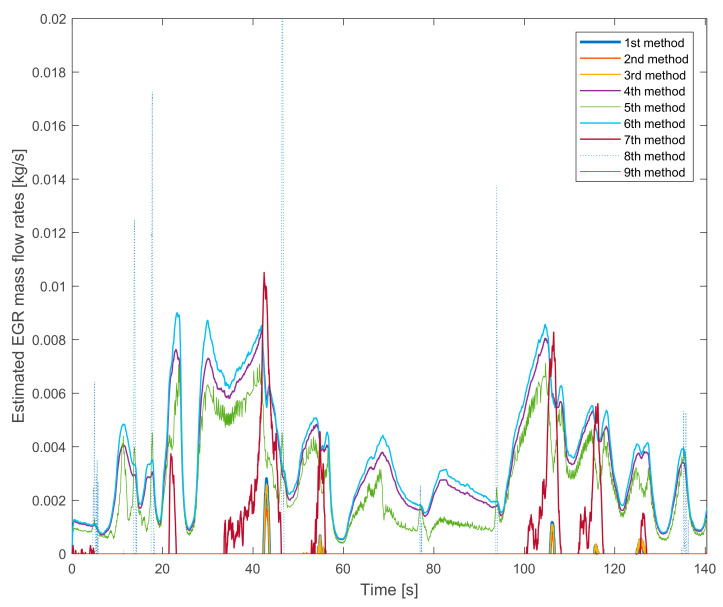
The results of the EGR estimation methods during the applied WHTC section without EGR.

Figure 11 presents significant errors of some EGR mass flow rate methods. In this measurement the EGR valves are closed. for example, there should not be any estimated EGR mass flow rates. 

The estimation methods that do not have this error (or negligible) are the 1st, the 2nd, the 3rd and the 9th methods. The estimation error is significant for the 4th, the 5th, the 6th, the 7th and the 8th methods. Thus, the oxygen mass fraction based EGR mass flow rate estimation seemed more suitable than the other methods.

## 7. Conclusions

Due to the increasingly strict emission norms for road vehicles, the technologies should be developed as well. In commercial vehicles, Diesel engines are still so widespread, to keep their harmful emission low, complex control systems are necessary. One of these is the EGR system, which can be mounted twice on a turbocharged engine: high pressure loop and low pressure loop.

In control synthesis for a dual loop EGR system, the issue of the EGR mass flow rate estimation frequently appears. In this paper, the possible measurement setups and the theoretical basics were presented for EGR mass flow rate estimation. Measuring the pressures, the temperatures, the mass fractions, the engine speed and the fuel consumption are useful for this aim. By the theoretical basics, finally, nine different estimation methods were created. Each of them needs a different sensor setup in the air path system.

It is important to emphasize that the research’s main aim was to find the best way to estimate the EGR mass flow rate. Most of the methods can probably be developed to reach better accuracy, for example, the differences between the methods can be decreased. Besides, the analysis of the sensors accuracy would give better results. Nonetheless, some conclusions can be drawn. The better EGR mass flow rate estimation methods can be selected for new, control oriented engine model development. Besides,

The main consequences are the followings:

- the oxygen mass fraction based EGR mass flow rate estimations provided consequently realistic results with low fluctuation,
- the volumetric efficiency based engine mass flow rate estimation provided acceptable signals on every engine load. It could be validated by the mass flow meter,
- the nitrogen-oxide mass fraction based EGR mass flow rate estimations provided higher values on low EGR rates. It can provide non-zero values in transient cycles with closed EGR valves, which obviously is an error. Probably a tunable multiplier can solve this issue,
- the air mass flow meter and mass fraction based methods gave too low EGR mass flow rates on low loads with very high EGR rates,
- the pressure drop and orifice equation based method provided different signals from the others. The tendencies of the EGR mass flow rate growth in function of the supporter exhaust brake closing was lower than the other signals. There are several articles where this method was validated, as was mentioned above [19,20,21]. In this case, this estimation method could be improved by further analysis,
- the mass conservation law based estimation method for the EGR mixer did not provide realistic signals. One of them had too high fluctuation due to the fuel consumption measurement signal. Besides, the other one usually provided too high values compared to the other estimation methods.

Evaluation of the presented EGR mass flow rate estimation methods:

- 1st method: verifiable, after some further research suitable for control aims,
- 2nd method: verifiable, after some further research suitable for control aims,
- 3rd method: non verifiable due to the non-logical behavior with high EGR rates,
- 4th method: non verifiable due to the non-zero value with closed EGR valves,
- 5th method: non verifiable due to the non-zero value with closed EGR valves,
- 6th method: non verifiable due to the non-zero value with closed EGR valves and to the non-logical behavior with high EGR rates,
- 7th method: non verifiable due to the non-zero value with closed EGR valves,
- 8th method: non verifiable, not suitable for the estimation due to the high fluctuation,
- 9th method: verifiable but recommended for further research to make it more accurate.

Considering the evaluation, the 1st and the partly the 2nd method is recommended in a mass fraction based control model. The common of them that they are based on oxygen mass fraction measurement, which sensors can be found in many serial engines.

By the concluded remarks, a development direction can be selected for mounting an appropriate sensor setup in the air path system of an internal combustion engine. The results can be useful for dual loop EGR analysis in commercial vehicle Diesel engines.

## Figures and Tables

**Table 1:** Parameters of the engine [27].

Type	Turbocharged Diesel, In-Line, 4 Cylinder
Maximum power	125 kW (2500 L/min)
Maximum torque	600 Nm (1200–1600 L/min)
Displacement	3.9 L
Stroke/bore ratio	1.176
Compression ratio	17.3
Injection system	direct injection, common rail
Maximum boost pressure	2.5 bar

**Table 2:** List of the applied sensor types.

Symbol	Parameter Name and Unit	Description	Type
*n_eng_*	Engine speed (1/s)	Engine speed sensor	IFM RO6345
*p*_1_	Compressor inlet pressure (Pa)	Compressor inlet pressure sensor	Gems 2200SG
*p*_2_	Intake manifold pressure (Pa)	Intake manifold pressure sensor	Gems 2200SG
*p*_3_	Exhaust manifold pressure (Pa)	Exhaust manifold pressure sensor	Gems 2200SG
*p*_5_	Pressure between the two exhaust brakes (Pa)	Compressor outlet pressure sensor	Gems 2200SG
*σ_air_*_0_	Intake fresh air mass flow rate (kg/s)	Intake air mass flow meter	Bosch HFM5
*σ_f_*	Fuel mass flow into the engine (kg/s)	Gravimetric fuel consumption meter	Energotest BDF700
		ECU of the engine + Control rapid prototyping and data acquisition (dSpace MicroAutoBox)	
*T*_2_	Intake manifold temperature (K)	Intake manifold temperature sensor	PT-100
*T*_3_	Exhaust manifold temperature (K)	Exhaust manifold temperature sensor	Ni-Cr-Ni
*T*_5_	Temperature between the two exhaust brake (K)	Turbine outlet temperature sensor	Ni-Cr-Ni
*x_O_*_2_	Oxygen gas mass composition rate (kg/kg)	Lambda sensor	Bosch LSU 4.9D
		Nitric oxide sensor	Continental UniNOx
*x_NOx_*	Nitrogen-oxide gas mass composition rate (kg/kg)	Nitric oxide sensor	Continental UniNOx

**Table 3:** Classification of the applied exhaust gas recirculation (EGR) mass flow rate estimation methods.

		Engine Mass Flow Rate Estimation		Intake Fresh Air Mass Flow Rate
		Engine Volumetric Efficiency	Engine Fuel Consumption	
**Mass fraction Changes**	**Oxygen**	1st method	2nd method	3rd method
	**NO_x_**	4th method	5th method	6th method
**Intake Fresh Air Mass Flow Rate**		7th method	8th method	-
**Pressure Drop**		9th method		-

**Figure A1:**
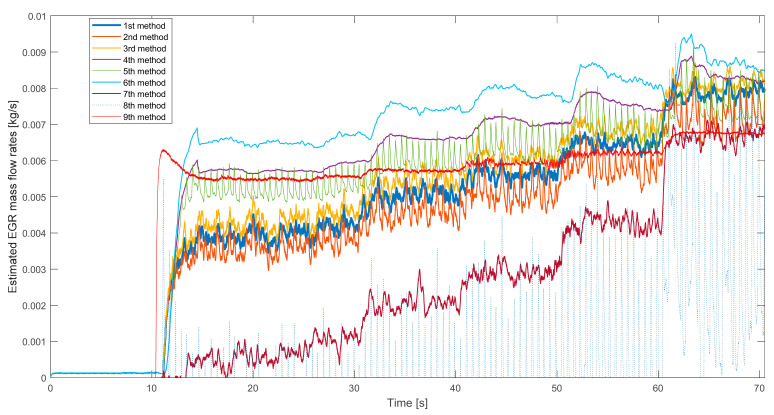
The estimated EGR mass flow rates with gradually closed exhaust brake. The HP EGR valve is opened, the engine runs on 1250 L/min and on 200 Nm.

**Figure A2:**
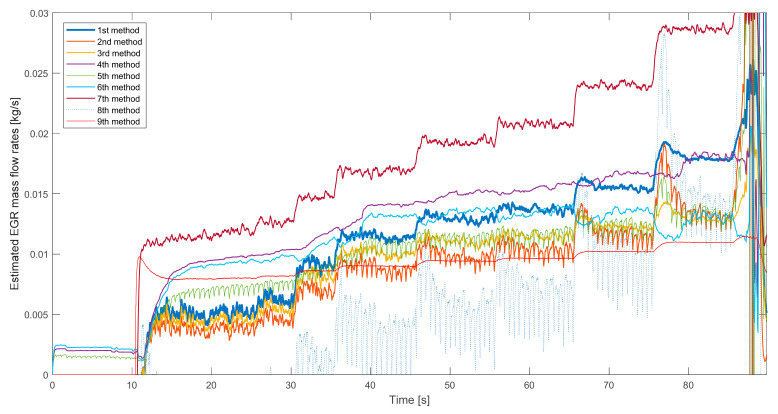
The estimated EGR mass flow rates with gradually closed exhaust brake. The HP EGR valve is opened, the engine runs on 1750 L/min and on 50 N m.

**Figure A3:**
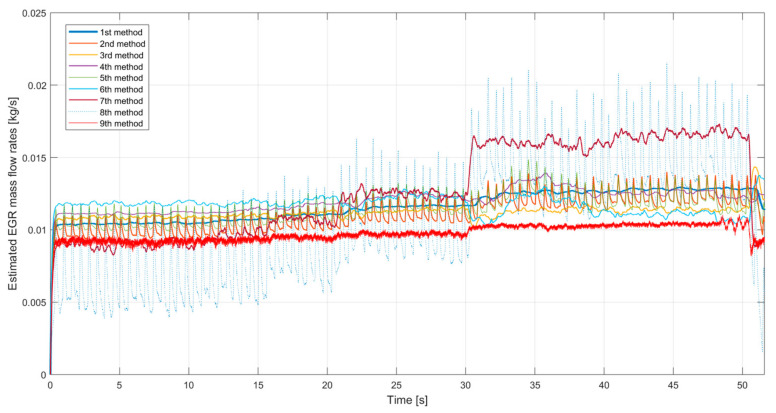
The estimated EGR mass flow rates with gradually closed exhaust brake. The LP EGR valve is opened, the engine runs on 1250 L/min and on 200 Nm.

**Figure A4:**
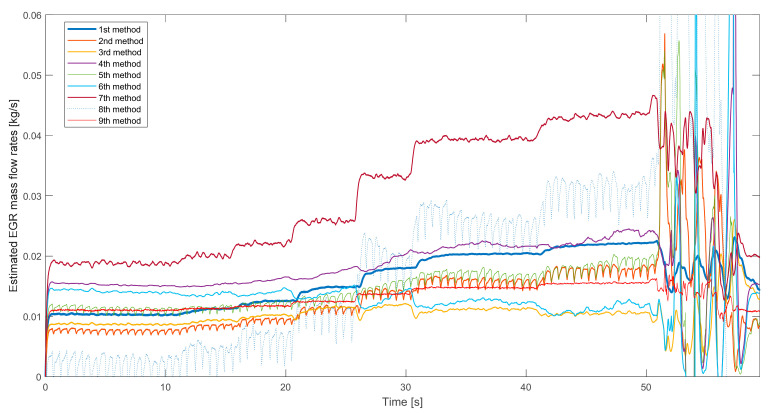
The estimated EGR mass flow rates with gradually closed exhaust brake. The LP EGR valve is opened, the engine runs on 1750 L/min and on 50 Nm.

## Appendix A

**Table A1:** Classification of the applied EGR mass flow rate estimation methods.

Symbol	Parameter Name and Unit
*A_HPL_*	Area of the HP EGR valve (m^2^)
*A_LPL_*	Area of the LP EGR valve (m^2^)
*A_eff_*	Area of the first exhaust brake (m^2^)
*ε*	Root-mean square error (**–**)
*η_vol_*	Volumetric efficiency of the engine (**–**)
*H_l_*	Diesel lower heating value (J/kg)
*K_L_*_0_	Stoichiometric air fuel ratio (**–**)
*κ*	Adiabatic exponent of air (**–**)
*n_eng_*	Engine speed (1/s)
*p_high_*	Higher pressure (Pa)
*p_low_*	Lower pressure (Pa)
*p*_1_	Compressor inlet pressure (Pa)
*p*_2_	Intake manifold pressure (Pa)
*p*_3_	Exhaust manifold pressure (Pa)
*p*_5_	Pressure between the two exhaust brakes (Pa)
*π*	Pressure ratio (**–**)
*π_crit_*	Critic pressure ratio (**–**)
*R*	Specific gas constant of air (J/kgK)
*σ_air_*_0_	Intake fresh air mass flow rate (kg/s)
*σ_eng_*	Mass flow rate through the internal combustion engine (kg/s)
*σ_f_*	Fuel mass flow into the engine (kg/s)
*σ_HPL_*	HP EGR mass flow rate (kg/s)
*σ_LPL_*	LP EGR mass flow rate (kg/s)
*σ_orifice_*	Mass flow rate through an orifice (kg/s)
*t*	Time (s)
*T_high_*	Higher temperature (K)
*T*_2_	Intake manifold temperature (K)
*T*_3_	Exhaust manifold temperature (K)
*T*_5_	Temperature between the two exhaust brake (K)
*V_d_*	Engine displacement (m^3^)
*x*_0_	Ambient gas mass composition rate (kg/kg)
*x*_2_	Intake manifold gas mass composition rate (kg/kg)
*x*_3_	Exhaust manifold gas mass composition rate (kg/kg)
*x_O_*_2,0_	Ambient gas oxygen mass composition rate (kg/kg)
*x_O_*_2,2_	Intake manifold oxygen gas mass composition rate (kg/kg)
*x_O_*_2,3_	Exhaust manifold oxygen gas mass composition rate (kg/kg)
*x_NOx,_*_0_	Ambient gas nitrogen-oxide mass composition rate (kg/kg)
*x_NOx,_*_2_	Intake manifold nitrogen-oxide gas mass composition rate (kg/kg)
*x_NOx,_*_3_	Exhaust manifold nitrogen-oxide gas mass composition rate (kg/kg)

## Appendix B

Estimation results in other stationary operation points.

## Appendix C

**Table A2:** Technical specification of the Gems 2200SG pressure sensors.

Specifications	Gems 2200SG
Pressure range	Vacuum to 400 bar
Proof pressure	2 × Full Scale (1.5 × Fs for 400 bar, >5000 psi
Long term drift	0.2% FS/year (non-cumulative)
Accuracy	0.25% FS typical (optional 0.15% FS)
Thermal error	0.5% FS typical (optional 1% FS)
Compensated Temperatures	−20 °C to +80 °C
Zero tolerance	1% of span
Span tolerance	1% of span
Response time	0.5 ms

**Table A3:** Technical specification of the Bosch HFM5 air mass flow meter sensor.

Specification	Bosch HFM5
Nominal supply voltage UN	14 V
Supply-voltage range UV	8...17 V
Output voltage UA	0...5 V
Input current IV	<0.1 A
Permissible vibration acceleration	≤150 ms–2
Time constant 1	≤15 ms
Time constant 2	≤30 ms
Temperature range	−40...+120 °C
Measuring range	12...640 kg/h
Accuracy	≤3%

**Table A4:** Technical specification of the PT100 temperature sensors.

Specifications:	Pt100 (100 Ohms @ 0 °C)
Construction:	Thin film, 10 mm tails
Temperature range:	−50 °C to +500 °C
Ice point resistance:	100 Ω
Fundamental interval (0 °C to 100 °C):	38.5 Ω (nominal)
Self-heating:	<0.5 °C/mW
Thermal response:	0.1 s
Stability:	±0.05%

**Table A5:** Technical specification of the NiCr-Ni temperature sensors.

Specification	Jacket Thermocouple, NiCr-Ni
Sheath material	Inconel 600
Permitted deviation	DIN EN 60 584-2: 1.5 °C or 0.004 (t) °C
Range of application	−220 °C +1150 °C
Response time	t90 = ca 3 s

**Table A6:** Technical specification of the Bosch LSU 4.9D lambda sensors.

Specification	Bosch Lambda Sensor LSU 4.9D
Application	Lambda 0.65 to ∞
Fuel compatibility	Diesel
Exhaust gas pressure	<4 bar
Exhaust gas temperature range (operating)	<930 °C
Exhaust gas temperature range (max.) for short time	<1030 °C
Max. vibration (stochastic peak level)	<1000 m/s2
Power supply H+ nominal	7.5 V
System supply voltage	10.8 V to 16.5 V
Heater power steady state	7.5 W
Heater control frequency	≥100 Hz
Nominal resistance of Nernst cell	300 Ω
Max current load for Nernst cell	250 µA
Signal output	Ip meas/Ua (AWS)
Accuracy at lambda = 0.8	0.80 ± 0.01
Accuracy at lambda = 1	1.016 ± 0.007
Accuracy at lambda = 1.7	1.70 ± 0.05

**Table A7:** Technical specification of the Continental UniNOx nitric oxide sensors.

Specification	UniNOx-Sensor
ZrO_2_-based multilayer sensor with 3 oxygen pumps	
Triple output signal (NOx, linear λ, binary λ)	
Supply voltage:	12 V/24 V
Measuring range:	NOx: 0–500 ppm or 0–1500 ppm
	lin. λ: 0.75 to air
	bin. λ: >0.75 V at λ = 0.9; <0.2 V at λ = 1.1
Accuracy:	NOx: at 100 ppm and 500 ppm: ± 10%; at 0 ppm: ± 10 ppm
	lin. λ: at λ = 1: ±6 (1000/λ) fresh
	bin. λ: 1.002 ± 0.008
